# A Distributed Cooperative Localization Strategy in Vehicular-to-Vehicular Networks

**DOI:** 10.3390/s20051413

**Published:** 2020-03-04

**Authors:** Minji Kim, Hong Ki Kim, Sang Hyun Lee

**Affiliations:** School of Electrical Engineering, Korea University, Seoul 02841, Korea; kimminji1013@korea.ac.kr (M.K.); istackcheese@korea.ac.kr (H.K.K.)

**Keywords:** autonomous vehicle localization, mm-wave connection, vehicle-to-vehicle network, message-passing algorithm

## Abstract

This work develops a distributed message-passing approach to cooperative localization for autonomous mobile vehicles that communicate via mm-wave wireless connection in vehicle-to-vehicle networks. Vehicles in the network obtain the measurement information about the relative distance and the angle of arrival from the mm-wave connections made with each other. Some vehicles may obtain knowledge about their absolute position information of different quality, for example, via additional localization feature. The main objective is to estimate the locations of all vehicles using reciprocal exchanges of simple information called a message in a distributed and autonomous way. A simulation is developed to examine the performance of the localization and navigation of vehicles under various network configurations. The results show that it does provide better positioning results in most cases and there are also several cases where the use of the cooperative technique adapts to design parameters such as accuracies of measurement equipment, and initial position estimates, that can affect the localization performance.

## 1. Introduction

With the recent introduction of 5th generation (5G) communication with low latency, large capacity, and high reliability characteristics, actual implementation of autonomous vehicles has been rendered more realistic [1]. Among the most important aspects of an autonomous vehicle, one very essential feature is the estimation of its position in a reliable and autonomous manner. This feature requires the robustness to inter-node connection failure, real-time updates, and high accuracy [2,3,4]. The localization of a group of vehicles can be conducted in a centralized manner; all vehicles’ locations can be estimated by a single computationally superior unit. While this approach alleviates computational demands of most vehicles in the network, its scalability has a limit since it is responsible for all processing steps such as collection, calculation, and distribution of the positioning information. Furthermore, the amount of devoted computation efforts scales directly with the population of vehicles in the network. Since the constraint on the scalability may cause significant challenges in applying the localization technique in an imminent large-scale vehicle-to-everything network, a distributed localization method where the positions of individual vehicles are identified locally and independently by themselves can be considered. A distributed method of the cooperative localization can adapt to a network of varying sizes because its local estimations involve only observations of a few neighboring units. In most cases, in order to obtain accurate location information, the global navigation satellite system (GNSS) is supported. However, its systems exploit essentially satellites and its effectiveness does not prove sufficient for autonomous driving in harsh environments with obstacles undermining the satellite signals, such as tunnels and undergrounds [5,6,7]. Therefore, one of the most promising alternatives for situations in which all vehicles do not receive GNSS signals is that vehicles in the wireless networks can independently infer their positions by exchanging local information [8] via mm-wave communication [2,9,10] which includes geometric analysis of received signals.

Localization problems have been studied steadily to meet these purposes using various techniques, such as maximum a posteriori (MAP) estimations [11,12], extended Kalman filters (EKF) [13,14,15], particle filters [16], and maximum likelihood (ML) estimations [17]. In References [11,12], factor graph models along with belief propagation algorithm are utilized to reduce complexity requirements in communication and computation and achieve joint cooperative localization and clock synchronization. In References [13,14,15], an extended Kalman filter technique is developed to achieve cooperative localization of large groups of mobile robots and road vehicles. In Reference [16], a particle filtering approach estimates the position and orientation of vehicles by combining the results from Global Positioning System-based absolute localization. The localization methods using extended Kalman filters and particle filters involve the data fusion of redundant readings of multiple sensors. In Reference [17] the ML estimation is combined with numerical optimization to solve the localization problem without external localization units. A representative distributed approach is based on sum-product algorithm over a wireless network (SPAWN) [18]. SPAWN shows good performance in terms of the accuracy since it efficiently calculates the posterior distribution of messages at each time instant. However, since the estimated distributions are nonparametric, their representation and evaluation are highly demanding for real-time updates. Recently, an new optimization technique based on distributed-alternating direction method of multipliers (ADMM) [19] is developed to lift a large amount of the computational cost in cooperative localization. However, ADMM updates the solution by using only average values of the probability distribution as position estimates, an extended technique that allows joint updates with the average and the accuracy for reliability adaptation for the position estimate is expected to improve the performance.

To this purpose, this work models a message-passing (MP) based technique that allows us to use all available measurement information for distributed cooperative localization. The ultimate goal aims at constructing a distributed algorithm capable of real-time positioning with high accuracy, turns out to be a completely new challenge compared with existing techniques [18,19]. In order to enhance the localization accuracy of a vehicular network, this work tries to update the accuracy with the average of the vehicle position. For a real-time deployment, this work proposes a novel modeling strategy that allows for the parametrization of complicated probability distributions for the position estimate and the its accuracy. In this technique, messages are calculated and exchanged cooperatively among neighboring vehicles to provide the estimate of the position with sufficient quality. Furthermore, additional reduction of communication overloads is available by employing a broadcasting policy to share the messages among adjacent vehicles. A simulation platform with graphical user interface (GUI) is developed to test the accuracy of the localization in real-time assessment for case studies. In practical circumstances including sensor noises, specific conditions are addressed for when the cooperative positioning becomes valid to improve the network-wide performance.

Major contributions of this work are summarized as follows:

- A mathematical formulation is constructed for vehicular self-localization and identifying vehicles’ relative positions based on a V2X network where all vehicles do not necessarily obtain the exact position information from peripheral devices.
- A novel message-passing framework is developed to determine the positions of all vehicles. The algorithm carries out the exchange of only the estimate and accuracy of the position to save computation costs spent to obtain the improved representation of messages. In addition, a broadcast protocol is designed to decrease message calculations and communication overloads for networking.
- A GUI simulation platform is developed to test the vehicular network based on 3GPP TR 37.885 specifications. The performance comparison among various distributed strategies is made to justify the localization performance of the proposed algorithm.

The rest of this paper is organized as follow: Section 2 presents the system model in a mm-wave connection based vehicular network. An MP algorithm is derived to handle the position estimates and their accuracies for cooperative localization in Section 3. Section 4 evaluates the performance of the proposed algorithm, and Section 5 concludes the paper.

## 2. System Model

### 2.1. Vehicular Network Model

This section introduces a system model for cooperative localization in a vehicle-to-vehicle (V2V) network. The vehicles in the network move along the road, and they can communicate with at least one of their neighbors. Among the vehicles, at least one vehicle, called an anchor, is capable of obtaining different quality measurements received from peripheral devices over other vehicles, called agents [18]. Let $x_{j}^{\left(t\right)}=\left[x_{1j}^{\left(t\right)},x_{2j}^{\left(t\right)}\right]$ and $x_{k}^{\left(t\right)}=\left[x_{1k}^{\left(t\right)},x_{2k}^{\left(t\right)}\right]$ be two-dimensional coordinate vectors of vehicle *j* and vehicle *k* at time *t*, respectively. Vehicles make measurements about their movement and communicate in mm-wave with surrounding vehicles to obtain the estimate about the relative distance and the angle of arrival (AoA) [2,20]. The relative distance measured by vehicle *j* from vehicle *k* at time *t* is given by

(1) $$\begin{array}{c} d_{kj}^{\left(t\right)}=∥x_{k}^{\left(t\right)}-x_{j}^{\left(t\right)}∥, \end{array}$$

where ∥·∥ is an Euclidean distance. Also, the AoA value measured by vehicle *j* from vehicle *k* is given by

(2) $$\begin{array}{c} \theta_{kj}^{\left(t\right)}=\tan^{-1}\frac{x_{2k}^{\left(t\right)}-x_{2j}^{\left(t\right)}}{x_{1k}^{\left(t\right)}-x_{1j}^{\left(t\right)}}. \end{array}$$

**Figure 1:**
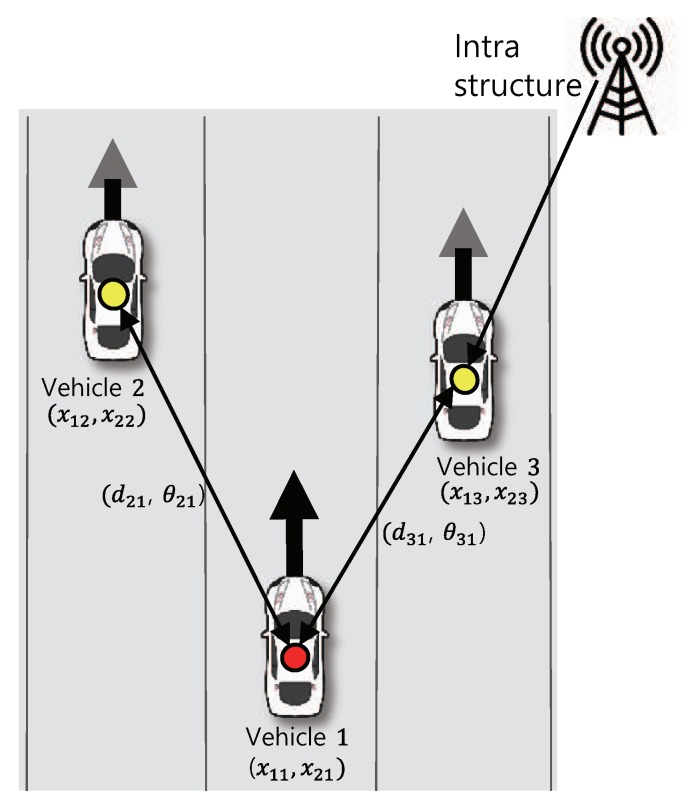
Cooperative localization in mm-wave vehicular network.

Figure 1 represents an example of the distributed vehicular network. There are three vehicles: two agent vehicles and a single anchor vehicle which obtains relatively improved position information from the network infrastructure. The vehicles communicate with each other and, in particular, vehicle 1 obtains the measurement about $\left(d_{i1}^{\left(t\right)},\theta_{i1}^{\left(t\right)}\right)\;\left(i=2,3\right)$ in mm-wave communication with vehicles 2 and vehicle 3.

To estimate the vector $x_{j}^{\left(t\right)}$ for positioning at time *t*, a Bayesian technique can be used to find the posterior probability distribution for $x_{j}^{\left(t\right)}$ from available information. For the information about the movement of vehicle *j*, vehicle *j* estimate its position denoted by $q_{j}^{\left(t\right)}$ only using the previous position $x_{j}^{\left(t-1\right)}$ and its noisy measurement of the speed of driving. Subsequently, the mobility model for vehicle *j* available to itself is characterized by a prior probability distribution denoted by $p(x_{j}^{\left(t\right)}|q_{j}^{\left(t\right)})$. Furthermore, the measurement model about the collection of parameters received from a surrounding vehicle *k* denoted by $w_{d_{kj},\theta_{kj}}^{\left(t\right)}$ is characterized by a likelihood function $p(w_{d_{kj},\theta_{kj}}^{\left(t\right)}|x_{j}^{\left(t\right)})$. With those probabilistic functions, the identification of the MAP estimate of $x_{j}$, denoted by ${\hat{x}}_{j}$, is pursued. For the MAP estimation, the marginal posterior $p(x^{\left(t\right)}|w_{d_{kj},\theta_{kj}}^{\left(t\right)},q_{j}^{\left(t\right)})$ is necessary and is represented as

(3) $$\begin{array}{c} p\left(x_{j}^{\left(t\right)}|w_{d_{kj},\theta_{kj}}^{\left(t\right)},q_{j}^{\left(t\right)}\right)=\frac{p\left(w_{d_{kj},\theta_{kj}}^{\left(t\right)}|x_{j}^{\left(t\right)}\right)p\left(x_{j}^{\left(t\right)}|q_{j}^{\left(t\right)}\right)}{p(w_{d_{kj},\theta_{kj}}^{\left(t\right)}|q_{j}^{\left(t\right)})}\propto p\left(w_{d_{kj},\theta_{kj}}^{\left(t\right)}|x_{j}^{\left(t\right)}\right)p\left(x_{j}^{\left(t\right)}|q_{j}^{\left(t\right)}\right). \end{array}$$

Several reasonable assumptions can be made in identifying the positions of vehicles: All vehicles move independently of each other and their movement are modeled in terms of Markov chains. In other words, the knowledge about the mobility obtained internally by each vehicle is independent and is not affected by other vehicles. Furthermore, the relative measurement results are also independent of each other, while the relative measurement results are only affected by the current condition of the vehicle.

### 2.2. Mobility Model

With the knowledge of the mobility model $p(x_{j}^{\left(t\right)}|q_{j}^{\left(t\right)})$, vehicle *j* predicts its position internally. Let $v_{j}\left(t\right)=\left[v_{1j}\left(t\right),v_{2j}\left(t\right)\right]$ denote the estimated velocities of vehicle *j* along the x-axis and the y-axis of the domain at time instant *t*. This can be modeled as a pair of random variables, since it is subject to corrupted measurement incurred by sensory hardware imperfection, which is characterized by noise variance ${\tilde{\sigma }}^{2}$. The interval between consecutive time instants is denoted by Δt. In addition, the mean and variance of the mobility model $p(x_{j}^{\left(t\right)}|q_{j}^{\left(t\right)})$ are given by an estimate vector $q_{j}^{\left(t\right)}$ and an accuracy vector $\varphi_{j}^{q}\left(t\right)$, which can be expressed, respectively, as

(4) $$\begin{array}{cc} q_{j}^{\left(t\right)} & =x_{j}^{\left(t-1\right)}+v_{j}\left(t\right)\Delta t, \end{array}$$

(5) $$\begin{array}{cc} \varphi_{j}^{q}\left(t\right) & =[\varphi_{1j}^{q}\left(t\right),\varphi_{2j}^{q}\left(t\right)]. \end{array}$$

Note that each component of the accuracy vector $\left[\varphi_{1j}^{q},\varphi_{2j}^{q}\right]$ corresponds to the variation of the velocity in the x-axis and the y-axis directions, respectively.

### 2.3. Measurement Model

Vehicles *j* uses measurement model $p(w_{d_{kj},\theta_{kj}}^{\left(t\right)}|x_{j}^{\left(t\right)})$ to estimate the relative distance and the AoA from neighboring vehicle *k*. The noisy measurement obtained by vehicle *j* about the distance to vehicle *k* is expressed as $w_{d_{kj}}^{\left(t\right)}=d_{kj}^{\left(t\right)}+n_{d_{kj}}^{\left(t\right)}$. The distance measurement noise $n_{d_{kj}}^{\left(t\right)}$ being a zero-mean Gaussian random variable with variance $\sigma_{n_{d}}^{2}$. Likewise, the noisy AoA measurement is given by $w_{\theta_{kj}}^{\left(t\right)}=\theta_{kj}^{\left(t\right)}+n_{\theta_{kj}}^{\left(t\right)}$. The AoA measurement noise $n_{d_{kj}}^{\left(t\right)}$ is also a zero-mean Gaussian random variable with variance $\sigma_{n_{\theta }}^{2}$. If vehicle *j* measures two parameters from vehicle *k*, likelihood function $p(w_{d_{kj},\theta_{kj}}^{\left(t\right)}|x_{j}^{\left(t\right)})$ for vehicle *j* is expressed as

(6) $$\begin{array}{c} p\left(w_{d_{kj},\theta_{kj}}^{\left(t\right)}|x^{\left(t\right)}\right)=p\left(w_{d_{kj}}^{\left(t\right)}|x_{j}^{\left(t\right)},x_{k}^{\left(t\right)}\right)p\left(w_{\theta_{kj}}^{\left(t\right)}|x_{j}^{\left(t\right)},x_{k}^{\left(t\right)}\right), \end{array}$$

where $p(w_{d_{kj}}^{\left(t\right)}|x_{j}^{\left(t\right)},\;x_{k}^{\left(t\right)})$ and $p(w_{\theta_{kj}}^{\left(t\right)}|x_{j}^{\left(t\right)},\;x_{k}^{\left(t\right)})$ are given, respectively, by

(7) $$\begin{array}{c} p\left(w_{d_{kj}}^{\left(t\right)}|x_{j}^{\left(t\right)},\;x_{k}^{\left(t\right)}\right)=\frac{1}{\sqrt{2\pi \sigma_{n_{d}}^{2}}}\exp\left\{-\frac{|w_{d_{kj}}^{\left(t\right)}-d_{kj}^{\left(t\right)}{|}^{2}}{2\sigma_{n_{d}}^{2}}\right\}, \end{array}$$

(8) $$\begin{array}{c} p\left(w_{\theta_{kj}}^{\left(t\right)}|x_{j}^{\left(t\right)},\;x_{k}^{\left(t\right)}\right)=\frac{1}{\sqrt{2\pi \sigma_{n_{\theta }}^{2}}}\exp\left\{-\frac{|w_{\theta_{kj}}^{\left(t\right)}-\theta_{kj}^{\left(t\right)}{|}^{2}}{2\sigma_{n_{\theta }}^{2}}\right\}. \end{array}$$

Note that these mobility and measurement distribution models enforce two different types of constraint functions in deriving a message-passing algorithm in the following section.

## 3. Proposed Message-Passing Algorithm

To develop a distributed algorithm via a message-passing framework, a factor graph [21] is introduced. The factor graph consists of function nodes and variable nodes. A function node is usually represented in a square, while a variable node is denoted in a circle in the graph. Each variable node is connected by an edge with its associated function node. In this problem, each variable node is associated with the coordinates of a vehicle, and two types of function nodes are introduced to reflect the internal mobility model and the measurement model. Since the measurement model is based on the information between each pair of adjacent vehicles, the corresponding function node has two connections to two variable nodes. On the other hand, a function node associated with mobility model is connected to a single variable node since the mobility model involves only its internal state of each vehicle. The messages are calculated using the sum-product rule [21] and are exchanged along connected edges. The repeated exchanges of the messages continue until all messages converge to respective fixed values.

The algorithm proceeds in three steps: First, a message which evidences the next position of a vehicle goes from a mobility function node to the corresponding variable node. The second-step message that contains the information about the relative distance and the AoA between each pair of adjacent vehicles also goes from a measurement function node to a variable node. The last message corresponding to the estimated current position of an individual vehicle goes back from the associated variable node to all neighboring function nodes. A single iteration consists of three steps of the message calculation. The iteration, indexed by *l*, of message transfers at a time instant is repeated until the value of messages stop changing. To be specific, the messages of vehicle *j* corresponding to three steps at a time instant are denoted by $\mu (x_{j}^{\left(t\right)},t)$, $\vec{\mu }\left(x_{j}^{\left(t\right)},l\right)$, and $\overset{\leftarrow }{\mu }\left(x_{j}^{\left(t\right)},l\right)$, respectively. Among those values, the last two message vectors are expressed as

(9) $$\begin{array}{cc} \vec{\mu }\left(x_{j}^{\left(t\right)},l\right)= & \left\{{\vec{\mu }}_{h_{1}}\left(x_{j}^{\left(t\right)},l\right),{\vec{\mu }}_{h_{2}}\left(x_{j}^{\left(t\right)},l\right),\ldots ,{\vec{\mu }}_{h_{n}}\left(x_{j}^{\left(t\right)},l\right)\right\}, \end{array}$$

(10) $$\begin{array}{cc} \overset{\leftarrow }{\mu }\left(x_{j}^{\left(t\right)},l\right)= & \left\{{\overset{\leftarrow }{\mu }}_{h_{1}}\left(x_{j}^{\left(t\right)},l\right),{\overset{\leftarrow }{\mu }}_{h_{2}}\left(x_{j}^{\left(t\right)},l\right),\ldots ,{\overset{\leftarrow }{\mu }}_{h_{n}}\left(x_{j}^{\left(t\right)},l\right)\right\},\;h_{1},h_{2},\ldots ,h_{n}\in N\left(j\right), \end{array}$$

where N(j) is a set of indices for neighboring vehicles that can communicate with vehicle *j*.

Consider the first-step message update rule defined with respect to the mobility model. Let $\mu (x_{j}^{\left(t\right)},t)$ be the message associated with internal mobility model for vehicle *j* at time *t*. According to the sum-product update rule, message $\mu (x_{j}^{\left(t\right)},t)$ is simply given by mobility probability distribution $p(x_{j}^{\left(t\right)}|q_{j}^{\left(t\right)})$. Since $p(x_{j}^{\left(t\right)}|q_{j}^{\left(t\right)})$ is simply represented by mean and variance, $\mu (x_{j},t)$ can be characterized with position vector $q_{j}^{\left(t\right)}$ and accuracy vector $\varphi_{j}^{q}\left(t\right)$, which are given, respectively, as

(11) $$\begin{array}{cc} q_{j}^{\left(t\right)} & ={\hat{x}}_{j}^{\left(t-1\right)}+v_{j}\left(t\right)\Delta t, \end{array}$$

(12) $$\begin{array}{cc} \varphi_{j}^{q}\left(t\right) & =[\varphi_{1j}^{q}\left(t\right),\varphi_{2j}^{q}\left(t\right)]. \end{array}$$

The second-step message update rule is associated with the measurement model which vehicle *j* obtains the distance and the azimuth from neighboring vehicle *k*. Message ${\vec{\mu }}_{k}\left(x_{j}^{\left(t\right)},l\right)$ is calculated by integrating the product of the measurement model function and the incoming message as

(13) $$\begin{array}{c} {\vec{\mu }}_{k}\left(x_{j}^{\left(t\right)},l\right)=\int p\left(w_{d_{kj},\theta_{kj}}^{\left(t\right)}|x_{j}^{\left(t\right)},x_{k}^{\left(t\right)}\right){\overset{\leftarrow }{\mu }}_{k}\left(x_{k}^{\left(t\right)},l\right)dx_{k}^{\left(t\right)}. \end{array}$$

Note here that likelihood functions $p(w_{d_{kj}}^{\left(t\right)}|x_{j}^{\left(t\right)},x_{k}^{\left(t\right)})$ and $p(w_{\theta_{kj}}^{\left(t\right)}|x_{j}^{\left(t\right)},x_{k}^{\left(t\right)})$ are highly nonlinear since $d_{kj}^{\left(t\right)}$ and $\theta_{kj}^{\left(t\right)}$ are nonlinear functions of coordinates of vehicle as in (1) and (2). Thus, the corresponding message can be evaluated only by numerical integration and is not simple to handle in a real-time manner.

**Figure 2:**
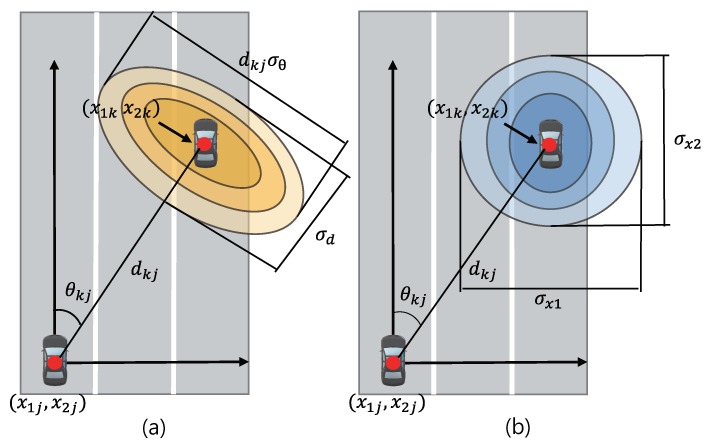
Measurement models: (**a**) original model (**b**) modified model.

To resolve unwieldy handling of this message, we introduce a new parameterized measurement model as shown in Figure 2, Figure 2a illustrates the confidence region of the measurement model where vehicle *j* measures $d_{kj}^{\left(t\right)}$ and $\theta_{kj}^{\left(t\right)}$ from neighboring vehicle *k*. Here, the challenge lies in that the axes of elliptical shapes representing the confidence region are slanted with respect to the direction of the movement of vehicle *j*. This significantly complicates to calculate the integration for the position of the vehicle.

**Figure 3:**
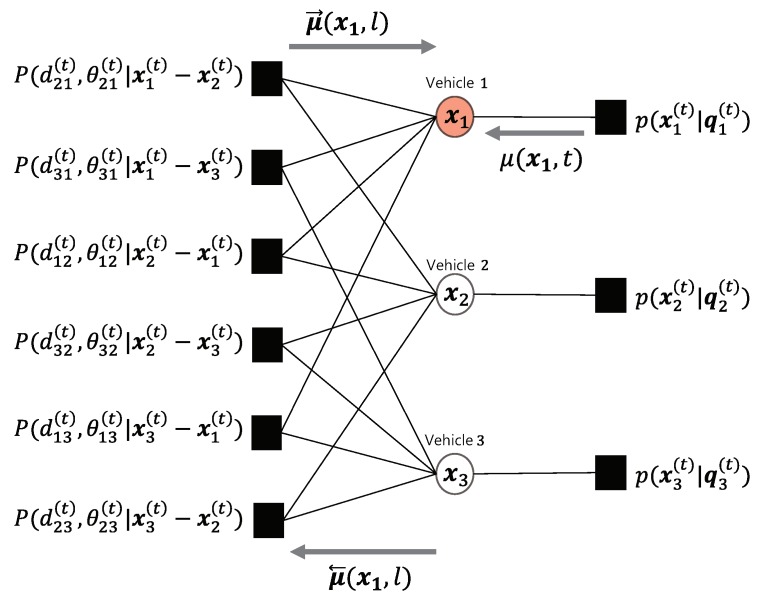
Graphical model associated with example in Figure 1.

To represent a new measurement model with respect to $x_{j}^{\left(t\right)}$ and $x_{k}^{\left(t\right)}$, the elliptical areas in Figure 2a are transformed into another elliptical shapes in Figure 2b, where horizontal and vertical axes are aligned toward the moving direction of vehicle *j*. By applying for this model with Cauchy-Schwarz inequality followed by some algebra, a new measurement model is characterized as a function of a two-dimensional coordinate difference vector of vehicle *j* and *k*. The resulting measurement model denoted by $p(d_{kj}^{\left(t\right)},\theta_{kj}^{\left(t\right)}|x_{j}^{\left(t\right)}-x_{k}^{\left(t\right)})$ is given by

(14) $$\begin{array}{c} p\left(d_{kj}^{\left(t\right)},\theta_{kj}^{\left(t\right)}|x_{j}^{\left(t\right)}-x_{k}^{\left(t\right)}\right)=\frac{1}{\sqrt{2\pi \varphi_{kj}^{w}\left(t\right)}}\exp\left(-\frac{{\left(w_{kj}^{\left(t\right)}-x_{j}^{\left(t\right)}+x_{k}^{\left(t\right)}\right)}^{2}}{2\varphi_{kj}^{w}\left(t\right)}\right), \end{array}$$

with estimate vector $w_{kj}^{\left(t\right)}=\left[w_{1kj}^{\left(t\right)},w_{2kj}^{\left(t\right)}\right]$ and accuracy vector $\varphi_{kj}^{w}\left(t\right)=\left[\varphi_{1kj}^{w}\left(t\right),\varphi_{2kj}^{w}\left(t\right)\right]$ are given by

(15) $$\begin{array}{c} w_{1kj}^{\left(t\right)}=-d_{kj}^{\left(t\right)}\cos\theta_{kj}^{\left(t\right)} \end{array}$$

(16) $$\begin{array}{c} w_{2kj}^{\left(t\right)}=-d_{kj}^{\left(t\right)}\sin\theta_{kj}^{\left(t\right)}, \end{array}$$

and

(17) $$\begin{array}{c} \varphi_{1kj}^{w}\left(t\right)=\sigma_{d_{kj}}^{2}\cos^{2}\theta_{kj}^{\left(t\right)}+{\left(d_{kj}^{\left(t\right)}\right)}^{2}\sigma_{\theta_{kj}}^{2}\sin^{2}\theta_{kj}^{\left(t\right)} \end{array}$$

(18) $$\begin{array}{c} \varphi_{2kj}^{w}\left(t\right)=\sigma_{d_{kj}}^{2}\sin^{2}\theta_{kj}^{\left(t\right)}+{\left(d_{kj}^{\left(t\right)}\right)}^{2}\sigma_{\theta_{kj}}^{2}\cos^{2}\theta_{kj}^{\left(t\right)}, \end{array}$$

respectively. Figure 3 shows the factor graph representing the new measurement model corresponding to the example of Figure 1. Now we can rewrite the message update equation as

(19) $$\begin{array}{cc} {\vec{\mu }}_{k}\left(x_{j}^{\left(t\right)},l\right) & =\int p\left(w_{d_{kj},\theta_{kj}}^{\left(t\right)}|x_{j}^{\left(t\right)},x_{k}^{\left(t\right)}\right){\overset{\leftarrow }{\mu }}_{j}\left(x_{k}^{\left(t\right)},l\right)dx_{k}^{\left(t\right)} \end{array}$$

(20) $$\begin{array}{cc} \; & =\int p\left(d_{kj}^{\left(t\right)},\theta_{kj}^{\left(t\right)}|x_{j}^{\left(t\right)}-x_{k}^{\left(t\right)}\right){\overset{\leftarrow }{\mu }}_{j}\left(x_{k}^{\left(t\right)},l\right)dx_{k}^{\left(t\right)}. \end{array}$$

Note here that the message sent from the measurement function node to variable node $x_{j}^{\left(t\right)}$ at iteration *l* evaluates the convolution with respect to $x_{k}^{\left(t\right)}$ instead of complex integral calculations with the original measurement model described with $d_{kj}^{\left(t\right)}$ and $\theta_{kj}^{\left(t\right)}$. The estimate and accuracy vectors of the resulting message are expressed as the sums of the estimate and accuracy vectors of two incoming messages respectively. Thus, each component of of message ${\vec{\mu }}_{k}\left(x_{j}^{\left(t\right)},l\right)$ can be inferred in a probability distribution given as

(21) $$\begin{array}{cc} {\vec{\mu }}_{k}\left(x_{1j}^{\left(t\right)},l\right) & \propto \frac{1}{\sqrt{2\pi \varphi_{1kj}^{\Delta }\left(l\right)}}\exp\left(-\frac{{\left(x_{1j}^{\left(l\right)}-\Delta_{1kj}^{\left(l\right)}\right)}^{2}}{2\varphi_{1kj}^{\Delta }\left(l\right)}\right), \end{array}$$

(22) $$\begin{array}{cc} {\vec{\mu }}_{k}\left(x_{2j}^{\left(t\right)},l\right) & \propto \frac{1}{\sqrt{2\pi \varphi_{2kj}^{\Delta }\left(l\right)}}\exp\left(-\frac{{\left(x_{2j}^{\left(l\right)}-\Delta_{2kj}^{\left(l\right)}\right)}^{2}}{2\varphi_{2kj}^{\Delta }\left(l\right)}\right), \end{array}$$

where components of estimate vector $\Delta_{kj}^{\left(l\right)}=\left[\Delta_{1kj}^{\left(l\right)},\Delta_{2kj}^{\left(l\right)}\right]$ and accuracy vector $\varphi_{kj}^{\Delta }\left(l\right)=\left[\varphi_{1kj}^{\Delta }\left(l\right),\varphi_{2kj}^{\Delta }\left(l\right)\right]$ are simply given, respectively, by

(23) $$\begin{array}{cc} \Delta_{1kj}^{\left(l\right)} & ={\hat{x}}_{1k}^{\left(l-1\right)}+w_{1kj}^{\left(t\right)} \end{array}$$

(24) $$\begin{array}{cc} \Delta_{2kj}^{\left(l\right)} & ={\hat{x}}_{2k}^{\left(l-1\right)}+w_{2kj}^{\left(t\right)}, \end{array}$$

and

(25) $$\begin{array}{cc} \varphi_{1kj}^{\Delta }\left(l\right) & =\varphi_{1k}^{\hat{x}}\left(l-1\right)+\varphi_{1j}^{w}\left(t\right) \end{array}$$

(26) $$\begin{array}{cc} \varphi_{2kj}^{\Delta }\left(l\right) & =\varphi_{2k}^{\hat{x}}\left(l-1\right)+\varphi_{2j}^{w}\left(t\right). \end{array}$$

Since the message with component distributions in (21) and (22) can be fully described using estimate vectors and accuracy vectors, it suffices to send them for the message transfer.

Finally, the message which a vehicle updates about its position and sends to a neighboring vehicle is calculated. The message sent from the variable node is updated using the sum-product update rule with all incoming messages from the measurement function nodes and a single message from the mobility function node. The resulting message update rule accounts for multiplying those incoming messages as

(27) $$\begin{array}{cc} {\overset{\leftarrow }{\mu }}_{k}\left(x_{j}^{\left(t\right)},l\right) & =\mu \left(x_{j}^{\left(t\right)},t\right)\prod_{h\in N(j)\backslash k}{\vec{\mu }}_{h}\left(x_{j}^{\left(t\right)},l\right), \end{array}$$

where N(j)\k is the set of neighboring vehicles that can send or receive the message from vehicle *j* except vehicle *k*. Since all incoming messages are represented with position estimate and accuracy vectors, the assumption of Gaussian shape distributions leads to their product being also of a Gaussian shape. Therefore, estimate and accuracy vectors of the outgoing message ${\overset{\leftarrow }{\mu }}_{k}\left(x_{j}^{\left(t\right)},l\right)$ can also be represented as the function of estimates and accuracy values of incoming messages. Each component of the resulting message is described in a probability distribution given by

(28) $$\begin{array}{cc} {\overset{\leftarrow }{\mu }}_{k}\left(x_{1j}^{\left(t\right)},l\right) & \propto \frac{1}{\sqrt{2\pi \varphi_{1j}^{\hat{x}}\left(l\right)}}\exp\left(-\frac{{\left(x_{1j}^{\left(t\right)}-{\hat{x}}_{1j}^{\left(l\right)}\right)}^{2}}{2\varphi_{1j}^{\hat{x}}\left(l\right)}\right) \end{array}$$

(29) $$\begin{array}{cc} {\overset{\leftarrow }{\mu }}_{k}\left(x_{2j}^{\left(t\right)},l\right) & \propto \frac{1}{\sqrt{2\pi \varphi_{2j}^{\hat{x}}\left(l\right)}}\exp\left(-\frac{{\left(x_{2j}^{\left(t\right)}-{\hat{x}}_{2j}^{\left(l\right)}\right)}^{2}}{2\varphi_{2j}^{\hat{x}}\left(l\right)}\right), \end{array}$$

where components of estimate vector ${\hat{x}}_{j}^{\left(l\right)}=\left[{\hat{x}}_{1j}^{\left(l\right)},{\hat{x}}_{2j}^{\left(l\right)}\right]$ and accuracy vector $\varphi_{j}^{\hat{x}}\left(l\right)=\left[\varphi_{1j}^{\hat{x}}\left(l\right),\varphi_{2j}^{\hat{x}}\left(l\right)\right]$ are calculated, respectively, as

(30) $$\begin{array}{cc} {\hat{x}}_{1j}^{\left(l\right)} & =\varphi_{1j}^{\hat{x}}\left(l\right)\left(\frac{q_{1j}^{\left(t\right)}}{\varphi_{1j}^{q}\left(t\right)}+\sum_{h\in N(j)\backslash k}\frac{\Delta_{1hj}^{\left(l\right)}}{\varphi_{1hj}^{\Delta }\left(l\right)}\right) \end{array}$$

(31) $$\begin{array}{cc} {\hat{x}}_{2j}^{\left(l\right)} & =\varphi_{2j}^{\hat{x}}\left(l\right)\left(\frac{q_{2j}^{\left(t\right)}}{\varphi_{2j}^{q}\left(t\right)}+\sum_{h\in N(j)\backslash k}\frac{\Delta_{2hj}^{\left(l\right)}}{\varphi_{2hj}^{\Delta }\left(l\right)}\right), \end{array}$$

and

(32) $$\begin{array}{cc} \varphi_{1j}^{\hat{x}}\left(l\right) & =\frac{1}{\frac{1}{\varphi_{1j}^{q}\left(t\right)}+\sum_{h\in N(j)\backslash k}\frac{1}{\varphi_{1hj}^{\Delta }\left(l\right)}} \end{array}$$

(33) $$\begin{array}{cc} \varphi_{2j}^{\hat{x}}\left(l\right) & =\frac{1}{\frac{1}{\varphi_{2j}^{q}\left(t\right)}+\sum_{h\in N(j)\backslash k}\frac{1}{\varphi_{2hj}^{\Delta }\left(l\right)}}. \end{array}$$

If a vehicle estimates its position and sends the corresponding message to neighboring vehicles, it calculates distinct forms of messages according to neighboring vehicles since the message update rule in (27) does not include the message coming from the direction of the message going out. However, as the network size scales up, the evaluation of all different messages incurs significant number of computations. Furthermore, for transmission of those messages, a sophisticated scheduling strategy that makes connections, transmits messages, and closes connections to respective neighbors is necessary, and its associated communication loads readily become critical for real-time operation. To resolve this scheduling challenge, identical messages are desired to send to all neighbors. If there are a sufficiently number of vehicles in the network, outgoing messages make little difference for all vehicles. Thus, the vehicles need to send a common message to surrounding vehicles to indicate its position. This makes little impact on the accuracy of cooperative positioning and boils down to a broadcast protocol for handling messages. Estimate vector ${\hat{x}}_{j}^{\left(l\right)}=\left[{\hat{x}}_{1j}^{\left(l\right)},{\hat{x}}_{2j}^{\left(l\right)}\right]$ and accuracy vector $\varphi_{j}^{x}\left(l\right)=\left[\varphi_{1j}^{\hat{x}}\left(l\right),\varphi_{2j}^{\hat{x}}\left(l\right)\right]$ of the probability distribution corresponding to a common message are described, respectively, as

(34) $$\begin{array}{cc} {\hat{x}}_{1j}^{\left(l\right)} & =\varphi_{1j}^{\hat{x}}\left(l\right)\left(\frac{q_{1j}^{\left(t\right)}}{\varphi_{1j}^{q}\left(t\right)}+\sum_{h\in N(j)}\frac{\Delta_{1hj}^{\left(l\right)}}{\varphi_{1hj}^{\Delta }\left(l\right)}\right) \end{array}$$

(35) $$\begin{array}{cc} {\hat{x}}_{2j}^{\left(l\right)} & =\varphi_{2j}^{\hat{x}}\left(l\right)\left(\frac{q_{2j}^{\left(t\right)}}{\varphi_{2j}^{q}\left(t\right)}+\sum_{h\in N(j)}\frac{\Delta_{2hj}^{\left(l\right)}}{\varphi_{2hj}^{\Delta }\left(l\right)}\right), \end{array}$$

and

(36) $$\begin{array}{cc} \varphi_{1j}^{\hat{x}}\left(l\right) & =\frac{1}{\frac{1}{\varphi_{1j}^{q}\left(t\right)}+\sum_{h\in N(j)}\frac{1}{\varphi_{1hj}^{\Delta }\left(l\right)}} \end{array}$$

(37) $$\begin{array}{cc} \varphi_{2j}^{\hat{x}}\left(l\right) & =\frac{1}{\frac{1}{\varphi_{2j}^{q}\left(t\right)}+\sum_{h\in N(j)}\frac{1}{\varphi_{2hj}^{\Delta }\left(l\right)}}. \end{array}$$

Note that, in fact, out of three-step message update rules, the first two update rules require only the information internally acquired within a vehicle. The last update rule, however, evaluates the messages that are externally exchanged with neighboring vehicles. It suffices for a vehicle to transmit only the messages calculated using (34)–(37). Thus, each vehicle calculates its position once with the common outgoing messages and broadcasts them to neighboring vehicles. By doing so, the total number of message calculations decreases, thereby lifting the communication burden. In addition, it has an advantage that vehicles can be learn simply all positions of surrounding vehicles.

**Figure 4:**
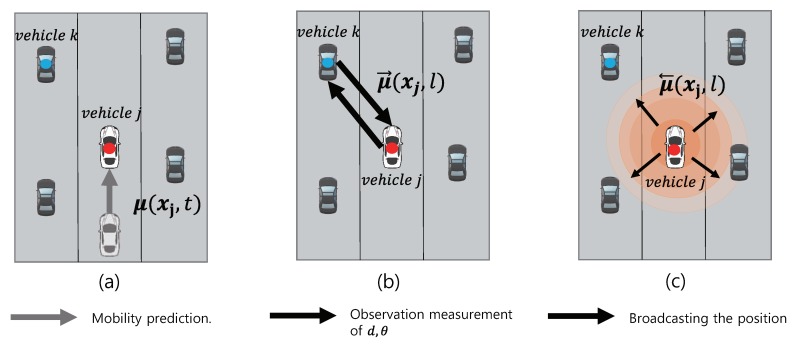
Three steps of proposed algorithm at time *t*: (**a**) mobility prediction step (**b**) measurement update step (**c**) message broadcast step.

In light of the structural discrepancy between the developed message-passing algorithm and its physical deployment of distributive positioning, additional consideration is required about handling of messages. The mm-wave technologies are used for conveying the messages computed by the message-passing operation. The signal strengths of the mm-wave signals can be measured by individual vehicles to acquire the estimates of the relative distances and AoAs of the neighboring vehicles. In practical implementation, the steps illustrated in Figure 4a–c are repeated while vehicles estimate their positions at time *t* via mm-wave technologies. In Figure 4a, vehicle *j* updates its new position estimate with the mobility model output. In Figure 4b, the signal strengths are measured to estimate the relative distance and AoA of the neighboring vehicle *k* and the function-to-variable messages in (23)–(26) are updated. Finally, in Figure 4c, vehicle *j* updates its position by collecting messages calculated in Figure 4a,b and, in turn, broadcasts it to surrounding vehicles. For practical deployment, the computation of messages in Figure 4a,b is carried out internally at each vehicle, while the communication with surrounding vehicles occurs only in Figure 4c. Algorithm 1 provides a summary of the proposed protocol for the distributed positioning algorithm.
**Algorithm 1** Simplified MP algorithm1:At time t=0 Initialization2:given ${\hat{x}}_{j}^{\left(0\right)}$, ${\hat{x}}_{k}^{\left(0\right)}$, ∀{j,k}, {j,k}∈N3:**for**t=1 to $T_{\max}$ **do** {time index}4: **for** nodes j∈N **do in parallel**5:  calculate the estimate and the accuracy of the message, $\mu (x_{j},t)$ in (11) and (12)6: **end for**7: **for** l=1 to $L_{\max}$ **do** {iteration index}8:  **for** j=1 to *N* **in parallel do**9:   calculate the estimate and the accuracy of the message $\vec{\mu }\left(x_{j},l\right)$ in (23)–(26)10:   calculate the estimate and the accuracy of the message $\overset{\leftarrow }{\mu }\left(x_{j},l\right)$ in (34)–(37)11:  **end for**12:  broadcast the estimate and the accuracy of the message $\overset{\leftarrow }{\mu }\left(x_{j},l\right)$13: **end for**14:**end for**

## 4. Performance Evaluation

In this section, we test the performance of the proposed MP algorithm (denoted by “SMP”) and compare with several algorithmic options, such as a different type of the message-passing algorithm [11] (denoted by “CSMP”), variational message-passing algorithm (denoted by “VMP”) [22], and extended Kalman filter (denoted by “EKF”) [13]. These algorithms run essentially in a distributed manner that requires similar levels of computational efforts and communication burdens, which allows fair comparison in the performance test. known to have similar computational costs for fair comparison.

### 4.1. Cooperative Localization Algorithm

Simulation has been conducted under vehicular network scenarios described in 3GPP TR 37.885 specification to verify the performance and strategy of the proposed algorithm. A V2V environment of 3GPP is a highway with 5 lanes. The width of the lanes is 3.5 m, and the moving distance is 2000 m. A single anchor is assumed to exist that can obtain its position with relatively improved accuracy with peripheral devices. The mobility model of each vehicle has the average speed of [0 kph, 72 kph] with variations ranging in [0.1 (kph)${}^{2}$, 1 (kph)${}^{2}$]. In the measurement model, the uncertainty with variation of 1 m and $3^{\circ }$ are incurred in *d* and θ respectively. Each vehicle measures its own speed every 100 ms and broadcasts a message every 10 ms. All vehicles obtain their initial positions corrupted by additive noises which cause vehicles to consider as if they are in a false lane. The simulation parameters are summarized in Table 1.

**Figure 5:**
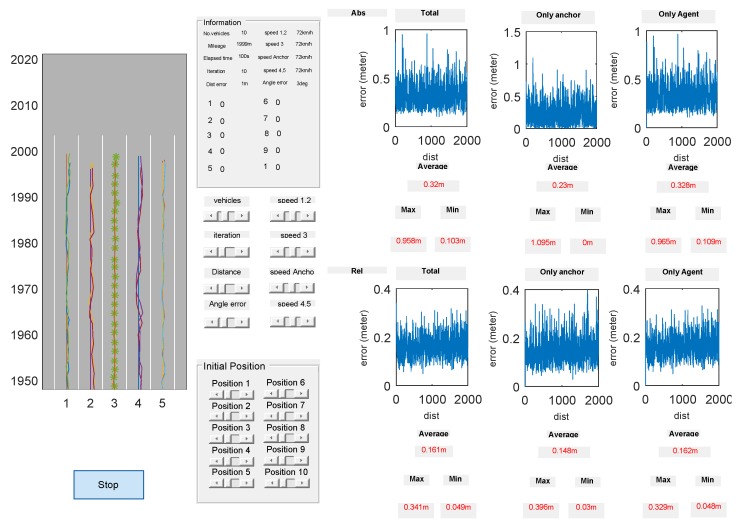
Graphical simulator.

A simulation platform that shows the performance of the proposed algorithm in real-time simulation has been developed as shown in Figure 5. The simulator consists of three domains: the visualization domain, the configuration domain, and the evaluation domain. The trajectory of vehicle positions is shown in the visualization domain. An anchor vehicle marked with ‘∗’ exists in the third lane. The configuration domain adjusts simulation environment parameters including the number of vehicles, the noise variance of *d* and θ, the starting position of vehicles, the speed of the vehicle and the number of iterations. The evaluation domain shows the real-time measurement of the maximum, minimum and average localization errors. Those errors are represented in two types of absolute and relative errors. The absolute error corresponds to the difference between the actual vehicle position and the estimated vehicle position, while the relative error indicates the error value associated with the relative distance between neighboring vehicles in the network. The absolute and relative error values of the vehicular network with agent vehicles and an anchor vehicle are plotted, respectively.

**Figure 6:**
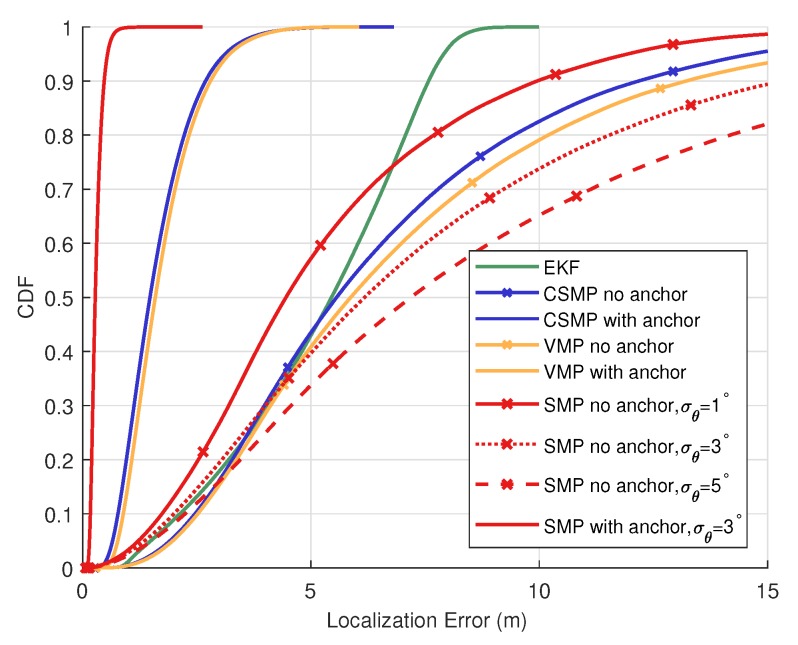
Performance comparison with various distributive techniques.

Figure 6 shows the performances of the proposed algorithm in comparison with several distributed algorithms in cases whether an anchor does or does not exist. The performance is presented with the cumulative distribution function (CDF) of the localization error with respect to observation parameters. SMP has superior performance over existing algorithms. CSMP has the degraded performance but outperforms other algorithms. In addition, VMP performs similarly but slightly worse than CSMP, while EKF shows additional performance degradation. If an anchor vehicle exists, the SMP shows improved performance with average error of 0.31 m, while CSMP, VMP and EKF have average errors of 1.64 m, 1.73 m and 5.16, respectively. Therefore, if at least one anchor exists in the network, even if the proposed algorithm has some level of the uncertainty in AoA, it can greatly improve the cooperative localization performance. On the other hand, in case where anchor vehicle lacks, the performance of SMP depends on the quality of the AoA. If the deviation of the AoA measurement is within $1^{\circ }$, SMP still has performance improvement over existing techniques. However, if the uncertainty in AoA measurement amounts up to $3^{\circ }$, SMP may be subject to a large absolute error. In case of the localization error value of 5 m, the deviation in the AoA measurement of $5^{\circ }$ causes the inversion of the localization error with respect to the unavailable AoA information measurement case. Since the distance between vehicles can be considered to be proportional to the localization error, the positioning error value can be regarded as the distance between vehicles. The farther vehicles are apart, the larger deviation on the information of the angles is received. Therefore, inaccurate information of θ can increase the localization error, thereby impairing the localization performance. To analyze the noise limit on the distance and AoA in the model based on 3GPP TR 37.885 specification, a limit of the standard deviation of the AoA is obtained from the Crammer-Rao Lower Bound with respect to the signal-to-noise ratio (SNR). The theoretical bound of the angle is 0.46° which is strictly smaller than 1°. According to this result, it is expected that SMP has relatively good performance with the error incurred in AoA available from reasonably practical measurement equipment.

**Figure 7:**
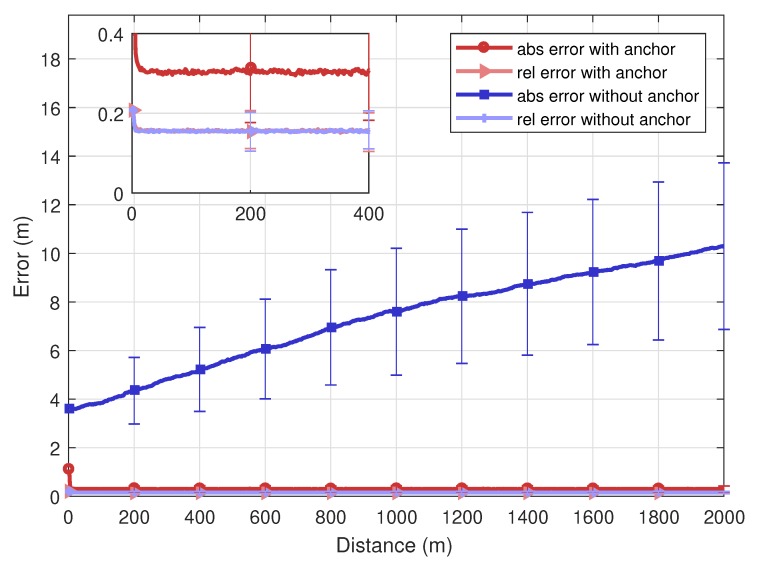
Error of SMP: with and without an anchor.

Figure 7 represents absolute and relative errors of the proposed algorithm with respect to the driving distance of vehicles. The total driving distance is 2000 m, and 10 vehicles exchange messages with each other. If no anchor exists, the absolute average error value continues to increase. Since a vehicle which has relatively high accuracy information of position lacks, the uncertainty of the positions continuously propagates over the network for their inaccurate operations while running along the road. On the other hand, if there is at least one anchor, the absolute error value is kept below 40 cm all the way to the driving distance of 2000 m. As a result, if only a small number of anchor vehicles is available, even a single one, the absolute positioning error of the entire network is greatly improved since messages that convey relatively accurate information helps locating all vehicles correctly. Meanwhile, the relative error maintains low regardless of the presence of the anchor vehicle. This indicates that the error is only affected by the measurement quality of observation parameters *d* and θ. Since the AoA information is available from other vehicles, the relative error value is kept as low as 20 cm on average. This means that all vehicles are affected by the same level of the bias in positioning. Therefore, there rarely happens a chance of dangerous situations caused by the collision among vehicles.

Table 2 shows trends of the absolute error with respect to the number of vehicles and the distance from an anchor vehicle to the group of agent vehicles. The total absolute error of the vehicular network increases as the distance increases, or as the number of vehicles in the network decrease. The minimum absolute error becomes down to 10 cm if a single anchor vehicle is close to 30 vehicles. On the other hand, the maximum error amount to 2.82 m if an anchor vehicle is apart from 5 agent vehicles. According to these observations, if the network size is large, accurate positioning is possible even if the anchor vehicle is relatively distant. By contrast, if the number of vehicles is small, an anchor is necessarily located nearby to maintain certain levels of the localization error. Therefore, this provides the information about the density of the anchor vehicle in the network required to guarantee the low level of the positioning error. For example, if the absolute positioning error value of the network should be kept within 1 m, there must be at least one anchor within a radius of 50 m for 5 vehicles, 150 m for 20 vehicles, 300 m for 30 vehicles in the network, and so on.

**Figure 8:**
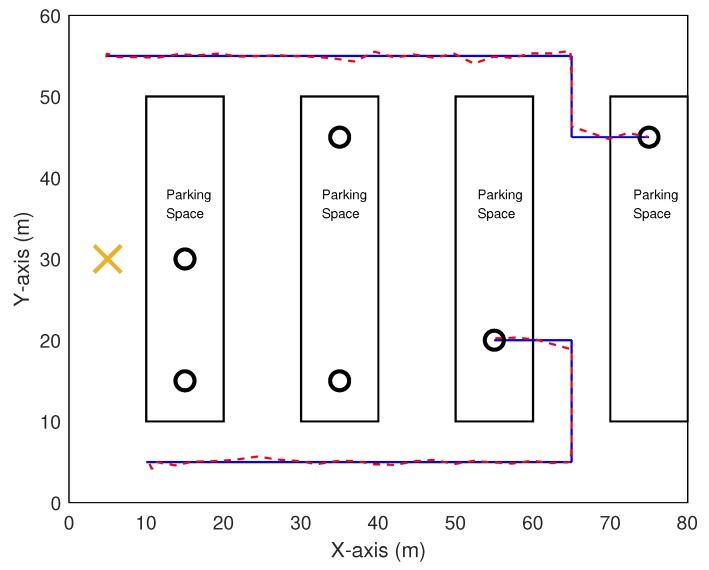
Trajectory of vehicles in an urban model.

Figure 8 illustrates additional cooperative localization results on a practical urban model with $80\times 60\;m^{2}$ rectangular region as in Reference [11]. In the model, there are a single anchor vehicle represented in a cross and six agent vehicles denoted in circles. Vehicles travel in low speed to reach their respective random destinations in parking spaces. The communication range is set to about 50 m. The leftmost anchor vehicle can access the position information of relatively improved quality. For clarity, estimated trajectories for two of seven vehicles are illustrated along with true trajectories. The estimated results look close to the truth with absolute error 0.74 m at worst in scenarios of such non-highway environments as well, which validates the developed SMP algorithm.

### 4.2. V2V Network Strategy with Corrupted Mobility Model

**Figure 9:**
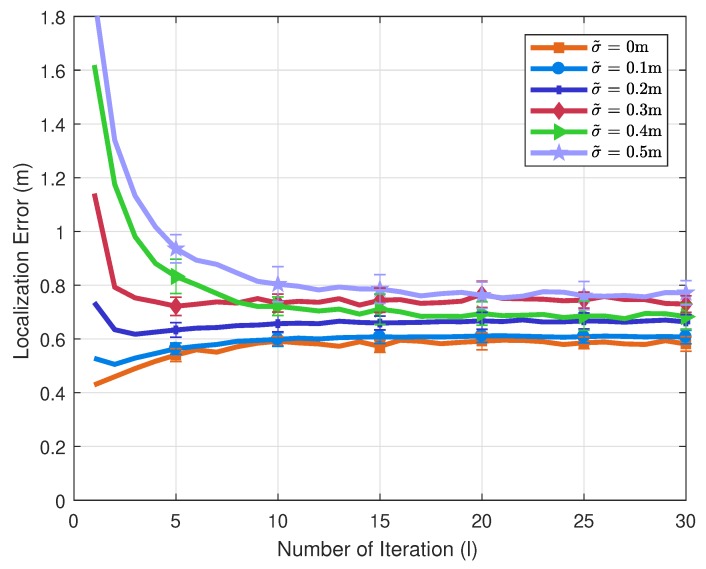
Average errors of 10 vehicles with respect to $\tilde{\sigma }$.

The uncertainty of the initial position of vehicles is already considered along with the communication strategy when measuring the relative distance and azimuth angle between vehicles. For practical deployment, the uncertainty caused by hardware imperfection is now addressed. This is referred to as a noisy mobility model. The uncertainty of measuring mobility measurement is a zero-mean Gaussian noise incurred in the existing mobility model in (11) and (12). Let $\tilde{\sigma }$ denote the standard deviation of the added noise. If a vehicle is in motion, there is some discrepancy between the actual speed of the vehicle and the speed displayed on the dashboard [23]. The uncertainty of the speed can be quantified with respect to the vehicular motion. Figure 9 shows the average absolute errors with respect to the number of message-passing iterations if $\tilde{\sigma }$ ranges from 0 m to 0.5 m. The simulation results indicate that cooperative positioning is essential if $\tilde{\sigma }$ is 0.2 m or greater, in that the localization error converges to the value strictly less than the initial value. If the $\tilde{\sigma }$ ranges below than 0.2 m, that is, the accuracy of the sensor is sufficiently high, cooperative positioning can be unnecessary, and the vehicles may prefer to estimate their positions based on their own mobility models. This indicates that in-vehicle networks of inexpensive hardware devices require cooperative positioning with surrounding vehicles.

We can also envision how much the positioning performance degrades in an unfavorable communication environment from Figure 9. The failure to the detection of message-conveying signals causes the loss of the message information along with the lack of the measurement information, thereby preventing from proceeding with another iteration of message-passing operations. In particular, in case of employing 10 iterations of message-passing operations, 20% packet loss, corresponding to 8 iterations, degrades the performance only by 2%. This shows that such an extreme packet loss results in negligible damages to the performance. Thus, this proves the robustness of the proposed algorithm in communication environments with typical qualities.

## 5. Conclusions

This paper proposes a message-passing algorithm that enables real-time distributed positioning in cooperation with surrounding vehicles if mm-wave communication is available. The proposed algorithm increases the possibility of the real-time communication by parameterizing complex messages into parameter pairs of estimate and accuracy. The broadcast strategy that allows for non-directional messages is employed so that the computational costs for radio resource allocation and scheduling tasks are relieved. According to the simulation results that have been analyzed with real-time errors, the performance of the proposed algorithm can be improved over existing distributed positioning algorithms.

In extensive numerical simulations under the highway environment provided from 3GPP TR 37.885 specification, the average absolute error below 40 cm and relative error below 20 cm are achieved, respectively. For various vehicular environments, the network size and the number of anchor vehicles are identified to maintain a certain level of the localization error. In addition, a proper condition that enables cooperative positioning is suggested under the uncertainty of vehicles’ mobility measurement. The analysis of the measurement equipment uncertainty indicates that cooperative positioning is essential if the standard deviation of the noise is greater than 0.2 m, whereas, if relatively accurate speed estimation is available, cooperative positioning may not be necessary. The proposed framework offers several future research directions for the deployment in a measurement model for urban area scenarios, such as in 3GPP TR 37.885 specification, with only non-line-of-sight (NLOS) measurement available and the experiments with actual mm-wave based radio technology.

## Figures and Tables

**Table 1:** Simulation parameters.

Parameter	Value
Network size $X_{1}\times X_{2}$ (m × m)	17.5 × 2000
The number of vehicles *N* (anchor/agent)	10 (1/9)
Average speed of vehicles v(t) (kph)	72
variance of speed $\varphi_{1}^{v},\;\varphi_{2}^{v},$ (kph)${}^{2}$	0.1, 1
Std. of distance measurement error $\sigma_{d}$ (m)	±1
Std. of AOA measurement error $\sigma_{\theta }$ (${}^{\circ }$)	±3
Discrete time period Δt (ms)	10
The number of Message iteration *l*	Δt/N
Initial position of N vehicles x(0)	0±0.1
The number of samples *M*	$10^{4}$
Std. of hardware noise $\left[{\tilde{\sigma }}_{1},{\tilde{\sigma }}_{2}\right]$ (m)	0, 0.1, 0.2, 0.3, 0.4, 0.5

**Table 2:** Absolute error trends depending on the number of vehicles and the radius of an anchor.

Number of Vehicles *N*	5	10	20	30
**0 m**	0.42 m	0.31 m	0.23 m	0.18 m
**50 m**	1.04 m	0.84 m	0.59 m	0.50 m
**100 m**	1.47 m	1.27 m	0.93 m	0.67 m
**150 m**	1.94 m	1.67 m	1.05 m	0.79 m
**200 m**	2.39 m	1.83 m	1.21 m	0.87 m
**250 m**	2.55 m	1.94 m	1.35 m	0.95 m
**300 m**	2.82 m	2.04 m	1.43 m	1.09 m
